# Feasibility and utility of a clinician dashboard from wearable and mobile application Parkinson’s disease data

**DOI:** 10.1038/s41746-019-0169-y

**Published:** 2019-09-25

**Authors:** Jordan J. Elm, Margaret Daeschler, Lauren Bataille, Ruth Schneider, Amy Amara, Alberto J. Espay, Michal Afek, Chen Admati, Abeba Teklehaimanot, Tanya Simuni

**Affiliations:** 1 Department of Public Health Sciences, Medical University of South Carolina, Charleston, SC 29425 USA; 2 The Michael J. Fox Foundation for Parkinson’s Research, New York, NY USA; 3 University of Rochester, Rochester, NY USA; 4 University of Alabama at Birmingham, Birmingham, AL USA; 5 University of Cincinnati Medical Center, Cincinnati, OH USA; 6 Advanced Analytics Department, Intel, Petach Tikva, Israel; 7 Northwestern University Feinberg School of Medicine, Chicago, IL USA

**Keywords:** Parkinson's disease, Health services, Prognostic markers

## Abstract

Mobile and wearable device-captured data have the potential to inform Parkinson’s disease (PD) care. The objective of the Clinician Input Study was to assess the feasibility and clinical utility of data obtained using a mobile health technology from PD patients. In this observational, exploratory study, PD participants wore a smartwatch and used the *Fox Wearable Companion* mobile phone app to stream movement data and report symptom severity and medication intake for 6 months. Data were analyzed using the Intel® Pharma Analytics Platform. Clinicians reviewed participants’ data in a dashboard during in-office visits at 2 weeks, 1, 3, and 6 months. Clinicians provided feedback in focus groups leading to dashboard updates. Between June and August 2017, 51 PD patients were recruited at four US sites, and 39 (76%) completed the 6-month study. Patients streamed 83,432 h of movement data from their smartwatches (91% of expected). Reporting of symptoms and medication intake using the app was lower than expected, 44% and 60%, respectively, but did not differ according to baseline characteristics. Clinicians’ feedback resulted in ten updates to the dashboard during the study period. Clinicians reported that medications and patient reported outcomes were generally discernable in the dashboard and complementary to clinical assessments. Movement, symptoms, and medication intake data were feasibly translated from the app into a clinician dashboard but there was substantial attrition rate over 6 months. Further enhancements are needed to ensure long-term patient adherence to portable technologies and optimal digital data transfer to clinicians caring for PD patients.

## Introduction

Parkinson’s disease (PD) patients are evaluated by their treating clinician for in-person visits infrequently, on average twice a year. These visits are typically short and can leave patients with limited time to discuss their symptoms and communicate questions about their disease and overall health. In addition, PD manifestations tend to be fluctuating, dependent on the time of the day and the relationship with the medication dose cycles, which may not be intuitive to patients. To overcome these challenges, a tool to capture the data in a more ecologically valid setting would be helpful for clinicians to inform therapeutic decisions.

The Michael J. Fox Foundation for Parkinson’s Research (MJFF) partnered with Intel Corporation to develop the *Fox Wearable Companion App* (FWC App), a mobile and wearable application for PD research. The FWC App is part of the Intel® Pharma Analytics Platform, an end-to-end artificial intelligence (AI) solution that enables remote monitoring and continuously captures clinical data from study subjects using a variety of sensors and wearable devices. Pairing a smartwatch and smartphone, accelerometer data collected from the smartwatch is transferred to the FWC App and transmitted to the cloud where advanced analytics and algorithms are applied on the data to generate various metrics such as activity level during waking and sleeping hours.^1^ Participants can also report various electronic patient-reported outcomes (ePROs) in the app, such as symptom severity, ON/OFF state and medication intake. MJFF has sponsored studies to support the development of this platform and the collection of sensor and ePRO data for analysis, curation and sharing with the scientific community. Studies have included the Levodopa Response Trial, the Parkinson@Home Study, and the study reported here, the Clinician Input Study (CIS-PD).^2,3^

The data collected by the FWC App may provide clinicians with a better understanding about the daily experiences of patients living with PD and the impact of their symptoms on their functional ability and quality of life.^4–6^ Real-world activity tracking and drug response reports could help clinicians recommend more personalized medication and lifestyle modifications. There are many challenges to incorporating app or wearable-derived information into clinical practice and clinical decision making. These include (1) a lack of validated, trusted data collection tools, (2) patient level of comfort and familiarity with novel technologies, and (3) an insufficient understanding of how to encourage long-term use and adoption of available technology options. This study focused on the distinct challenge of a lack of a widely accepted, user-friendly platform to report this type of data to clinicians in an easily understandable interface. CIS-PD was launched to assess the feasibility of use of the FWC app by PD patients over a 6-month period, to evaluate the utility of app-derived data to inform clinical decision making, and to iteratively develop and optimize a dashboard for data reporting with direct input from clinicians over the course of the study.

## Results

### Recruitment and retention

The study recruited 51 PD participants between June and August 2017 across the four study sites. The participants demographic and clinical characteristics are listed in Table 1. A total of 39 (76%) participants completed the full 6-month study. Reasons for early termination included: 3 participants did not like the watch (6%), 4 participants discontinued due to medical reasons unrelated to the study (8%), 2 participants due to time constraints (4%), and 3 participants for unknown reasons (6%).

**Table 1:** Baseline Characteristics of PD Patients overall and by compliance status

	Total PD participants *N* = 51	Low compliance *n* = 17	Medium compliance *n* = 17	High compliance *n* = 17	*P* value
Average compliance, mean (SD)	66% (34%)	28% (16%)	68% (10%)	102% (16%)	—
Enrolling site					0.06
Northwestern Memorial Hosp, Chicago	16 (31%)	6 (35%)	4 (24%)	6 (35%)	
Strong Memorial Hospital, Rochester	12 (24%)	1 (6%)	4 (24%)	7 (41%)	
University of Alabama Hosp, Birmingham	13 (25%)	8 (47%)	3 (18%)	2 (12%)	
University of Cincinnati Medical Center	10 (20%)	2 (12%)	6 (35%)	2 (12%)	
Male sex, *N* (%)	29 (57%)	9 (53%)	9 (53%)	11 (65%)	0.82
White, *N* (%)	46 (90%)	14 (82%)	16 (94%)	16 (94%)	0.60
Age, mean (SD)	61.9 (10.5)	58.6 (11.3)	62.5 (9.5)	64.6 (10.4)	0.07
Education, *N* (%)					0.61
High school	3 (6%)	1 (6%)	0 (0%)	2 (12%)	
Some college/associates	6 (12%)	0 (0%)	3 (18%)	3 (18%)	
Bachelors	19 (37%)	8 (47%)	6 (35%)	5 (29%)	
Masters/doctorate/professional degree	23 (45%)	8 (47%)	8 (47%)	7 (41%)	
Employment, *N* (%)					0.74
Working	20 (39%)	7 (41%)	6 (35%)	7 (41%)	
Retired	28 (55%)	8 (47%)	11 (65%)	9 (53%)	
Disabled	3 (6%)	2 (12%)	0 (0%)	1 (6%)	
Has a regular caregiver, *N* (%)	14 (27%)	5 (29%)	6 (35%)	3 (18%)	0.63
Skill level with electronic, web-based interfaces					0.35
Novice	3 (6%)	2 (12%)	1 (6%)	0 (0%)	
Intermediate	12 (24%)	5 (29%)	4 (24%)	3 (18%)	
Advanced	9 (18%)	3 (18%)	2 (12%)	4 (24%)	
Expert	27 (53%)	7 (41%)	10 (59%)	10 (59%)	
Years since symptom onset, mean (SD)	8.5 (5.0)	8.8 (5.4)	10.3 (5.7)	6.4 (3.1)	0.13
Years since diagnosis onset, mean (SD)	7.1 (4.8)	7.1 (5.4)	8.9 (5.3)	5.4 (3.1)	0.19
Hoehn and Yahr, mean (SD)	2.0 (0.4)	2.1 (0.6)	2.1 (0.4)	1.9 (0.2)	0.64
MDS-UPDRS part 1, mean (SD)	10.1 (4.9)	9.5 (4.0)	10.5 (5.4)	10.3 (5.3)	0.90
MDS-UPDRS part 2, mean (SD)	9.7 (5.5)	9.1 (6.2)	10.5 (5.7)	9.7 (4.6)	0.58
MDS-UPDRS part 3, mean (SD)	23.6 (11.4)	23.6 (12.0)	24.8 (13.2)	22.4 (9.3)	0.95
Fluctuators, *N* (%)	14 (27%)	6 (35%)	4 (24%)	4 (24%)	0.79
PDQ-39 summary, mean (SD)	17.9 (10.4)	14.7 (8.1)	21.5 (11.9)	17.5 (10.1)	0.17
MOCA ≥26, *N* (%)	38 (75%)	12 (71%)	12 (71%)	14 (82%)	0.78

*P*-values are two-sided and are from Kruskal–Wallis test for ordinal and continuous variables and Fisher’s exact test for categorical and binary variable. “Low”, “Medium”, and “High” compliance were defined by the tertiles of individuals’ average of the following: percent of hours streamed, percent of PRO reports, and percent of medication reports

### Feasibility of use of the wearable device and app

**Fig. 1:**
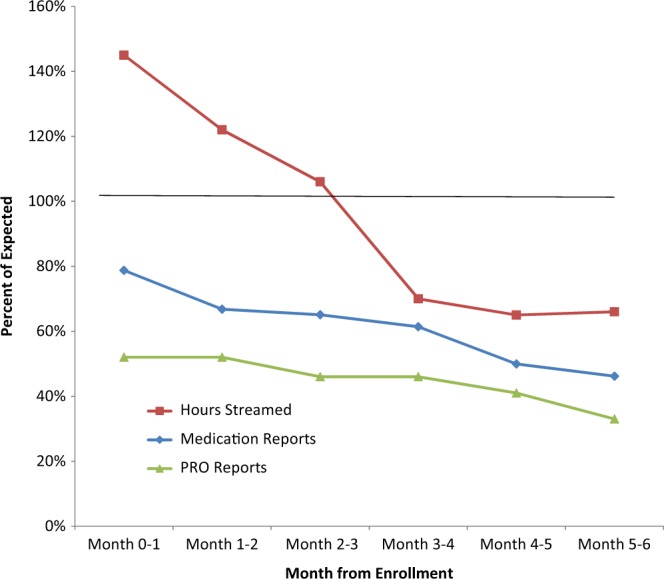
Percent of expected data streaming, medication reports, and PRO reports. Expected 12 h of data streamed × 25 days per month, three PRO reporting instances per day, and medication per schedule. If a patient withdrew consent or was lost to follow-up then streaming/reporting was not expected after the date of study withdrawal. These data were based upon 51 PD participants at month 0–1, 49 at month 1–2, 46 at month 2–3, 46 at month 3–4, 44 at month 4–5, 43 at month 5–6

Figure 1 shows the percent of expected data streaming, medication reports, and PRO reporting instances over time. The total number of hours of accelerometer data streamed from participants was 83,432 (91% of expected). The actual number of days in which 12 or more hours were streamed was fewer than expected (21, 17, 15, 10, 9, and 8 days on average in months 1, 2, 3, 4, 5, and 6, respectively, rather than the 25 days expected). Thus, in the first 3 months of study participation, PD participants streamed more hours of data than expected, with participants streaming >12 h on compliant days. Due to technical issues and support, streaming was lower than expected in October and November 2017, which were resolved at the end of November. Technical issues were mostly device connectedness and pairing issues between phone and watch. Regardless of this, the number of hours streamed decreased over time (145% during the first month versus 66% of expected during the 6th month). Overall, participants reported medication intake in 41,961 instances (60% of expected). Participants were asked to rate symptom severity (for up to eight types of symptoms) three times per day, but reporting instances were lower than expected, 44% overall, with an average of 8 daily reports of symptom severity. Overall, the average compliance was 66% (SD = 34%). Compliance level was similar across demographics and PD characteristics (MDS-UPDRS, MOCA, PDQ-39), although there was a marginal difference by age and enrolling site, which did not reach statistical significance (Table 1).

### Utility of clinician dashboard

**Fig. 2:**
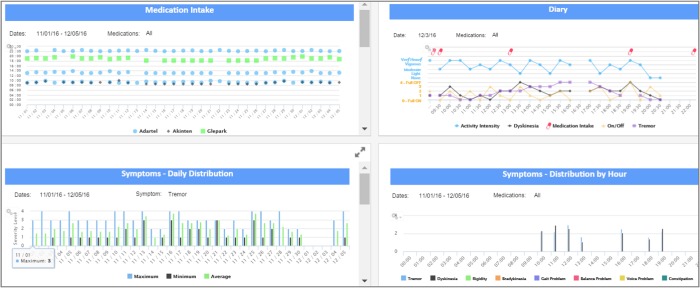
Clinician dashboard of displays for an individual PD patient. The clinician dashboard displays medication intake by drug name, a digital ON/OFF diary (ePRO), and daily/hourly symptom severity ratings (ePRO) for a given date range

Feedback on ways to make the dashboard easier to interpret and support clinical decision making were obtained from five clinician focus group sessions and resulted in ten dashboard updates during the course of the study, with about 4–6 clinicians attending each focus group. This included the creation of separate displays for ePRO and sensor-derived data display, the expansion of the Y-axis in certain displays for easier data comprehension, the addition of markers for medication intake across ePROs and sensor-derived data displays, updates to descriptive text and the addition of “info” buttons describing each display (Fig. 2). Clinicians also provided feedback that was not able to be incorporated over the course of the study, which has been included in Supplementary Table 1.

The final version of the dashboard was released on October 30, 2017. Based on this final version, the most beneficial component of the dashboard to clinicians was medication compliance followed by the severity of ePRO symptoms. Activity level and nighttime activity, both sensor-derived data points, were the least beneficial component for clinicians, but still supported the clinical assessment two-thirds of the time. The final iteration of the dashboard was used to display data for 49 patients across all sites. The final iteration of the dashboard provided clinicians with varying degrees of discernibility and utility to inform clinical decision making, depending on the item (medication compliance, symptom severity, tremor, etc.) as shown in Table 2. Using the final version of the dashboard, clinicians preferred the hourly displays of most components, except for the nighttime activity component for which daily displays were preferred. In the final version of the dashboard, more items were able to be discerned with a higher consistency and were supportive of the clinical assessment as compared with the first version of the dashboard.

**Table 2:** Utility of dashboard data in clinical care

	Able to discern in dashboard	How did these data assist you in your clinical assessment?		
		Contradicted	Neither here nor there	Supported
First version of the dashboard^a^				
Medication compliance	91%	32%	4%	65%
Symptom severity	64%	3%	20%	78%
Tremor	61%	15%	13%	72%
Gait impairment	50%	16%	22%	63%
Constipation	45%	7%	10%	83%
Dyskinesia	47%	24%	17%	59%
Activity level	88%	18%	15%	67%
Sleep dysfunction	22%	0%	21%	79%
Last version of the dashboard^b^				
Medication compliance	80%	7%	7%	85%
Symptom severity	76%	8%	8%	85%
Tremor	76%	10%	8%	82%
Gait impairment	67%	17%	9%	74%
Constipation	67%	9%	3%	88%
Dyskinesia	57%	28%	0%	72%
Activity level	61%	23%	10%	68%
Sleep dysfunction	18%	22%	11%	67%

^a^Percent of 67 assessments taken from 38 PD participants assessed in office using the first version of the clinician dashboard available from study start to August 23, 2017

^b^Percent of 51 assessments taken from 49 PD participants assessed in office using the last version of the clinician dashboard made available on or after October 30, 2017 until the end of the study

### Validation of ePRO reports

The correlations between MDS-UPDRS part 2 items and ePROs (averaged across the week prior to the in-office visit) were moderate. Spearman rho ranged from 0.55 to 0.74 for speech, 0.43 to 0.54 for balance, and 0.52 to 0.84 for tremor, where the ranges are the minimum and maximum correlation coefficient by visit (Table 3). The weighted Kappa’s were all <0.70, except for tremor (weighted Kappa 0.73) at 6 months.

**Table 3:** Correlation between PROs reported in app and MDS-UPDRS part 2 items

Symptom	Visit	Spearman correlation coefficient (two-sided *p*-value)	Kappa (weighted)	Kappa 95% CI	
Balance/walking	2 weeks	0.47 (0.0033)	0.35	0.13	0.56
	1 month	0.43 (0.0064)	0.27	0.07	0.48
	3 months	0.54 (0.0015)	0.38	0.15	0.62
	6 months	0.45 (0.0334)	0.35	0.07	0.63
Tremor	2 weeks	0.55 (0.0005)	0.38	0.19	0.58
	1 month	0.52 (0.0007)	0.41	0.2	0.61
	3 months	0.55 (0.0007)	0.38	0.15	0.62
	6 months	0.84 (< 0.0001)	0.73	0.54	0.92
Voice/speech	2 weeks	0.55 (0.0021)	0.31	0.08	0.53
	1 month	0.65 (< 0.0001)	0.39	0.17	0.6
	3 months	0.74 (< 0.0001)	0.54	0.34	0.74
	6 months	0.57 (0.0176)	0.35	0.08	0.62

## Discussion

Consistent with previous reports,^6–8^ our study supports the feasibility of remote data collection from people with PD. In general, PD participants were able to stream data from the watch and report symptoms and medication intake using the app. However, participants often skipped days, and the amount of streaming and reports declined over time, specifically after the first 3 months. Nearly a quarter of participants did not complete the whole 6 months. That is an important observation that will have to be taken into account in the design of the future long-term technology based monitoring. The reasons for this drop in compliance are likely multifactorial and could include study fatigue, decline in the novelty of the study technology, device-specific issues, and technical limitations. A longitudinal decline in use is common with such technologies (e.g., mPower app), and approaches to encourage continued use of this app will be needed (e.g., personalized feedback, community interactions, etc).^9^ There were no notable differences in baseline characteristics that explained compliance, though, perhaps surprisingly, the group with the highest compliance was somewhat older on average. The number of expected daily ePROs reported in the app (3 times per day for symptoms, all medications) and number of expected hours streamed was fairly high overall, and may be responsible for the reduced usage, or fatigue, over time. Our study did not include a survey to participants regarding reasons for study termination, and future studies should incorporate such feedback-collection mechanisms examine reasons for non-compliance over time (especially for studies longer than 3 months).

Findings from this study primarily focused on the ePRO data collected through the app and how that informed clinical judgment. While wearable sensor-derived data (activity level during day and night) were collected, they were not the focus of this analysis because of the exploratory nature of the algorithms used. Raw, sensor-level accelerometry data from the Apple Watch, along with clinical assessments and scales collected during study visits will be made available to the research community for future analyses.

A novel aspect of this study was the process of clinicians’ review of the app data, and subsequent iterative development of the dashboard tool during the course of the study period. Using feedback from clinicians based upon their experiences using the tool, ten dashboard modifications were made. Frequently, developers do not engage clinicians in the technology development process and will simply present the final prototype for clinical testing. This study demonstrated that clinicians should be involved from the earliest stages of the development of such platforms, both for clinical or research end-use. With the final version of the study dashboard tool, clinicians reported that medication compliance and symptom severity were generally discernable and supportive of the clinical assessments made in-office. Of note, dyskinesia (as reported through ePROs), activity level and sleep dysfunction (both sensor-derived data points), had the highest percent of disagreement with the clinician assessment, but as the read out for activity level and sleep dysfunction were based on an exploratory algorithm, this is not too surprising.

Another interesting observation is that there was only modest correlation between ePRO reports and the in-office MDS-UPDRS items, despite the fact that both are patient-reported and assessed the same contents. The discrepancy might reflect patient’s PRO underreporting and limitations of recall over the past week^10^ versus real-time reports collected within the app, which would highlight the importance of real-time data collection. Real-time patient-reporting on symptom severity will be crucial for future analysis of the CIS-PD sensor data for developing clinically meaningful digital measures of PD.

While the technology used in this study did involve some non-motor symptoms, it mostly focused on motor aspects of PD. Given the importance of non-motor symptoms on the quality of life of PD patients, future versions ought to consider a broader spectrum of non-motor ePROs, as is being developed elsewhere (e.g., mPower). Motor disability can impact utilization of devices and apps, however, we did not find disease severity to be a predictor of compliance. This is possibly because overall our cohort was representative of a milder disease, or perhaps a limitation of the sample size. Future studies will need to address feasibility across a wider spectrum of disease severity.

In conclusion, data collection from mobile and wearable technologies is feasible and can aid clinicians’ understanding of their patients’ daily experiences and inform their clinical care. Future studies utilizing technology based ePROs data collection should carefully assess the frequency and intensity of reporting and streaming requirements for patients and examine ways to increase long-term compliance. While clinicians’ involvement in the development of this type of clinical or research tools is important, patient’s involvement may be essential to ensure a compelling tool to suit their needs and increase adherence. Data collected from patients over long periods of time in real-world, natural settings may reveal new insights about individualized disability and inform tailored clinical care of PD patients.

## Methods

### Setting

CIS-PD was sponsored by MJFF and conducted across four US sites: Northwestern University, the University of Cincinnati, the University of Rochester, and the University of Alabama at Birmingham. Each site had local Institutional Review Board (IRB)/Research Ethics Board (REB) approval, and all participants signed informed consent.

PD participants were men and women aged 18 years or older with Hoehn and Yahr stage 1 to 3. In order to be eligible for the study, participants had to be able to attend research visits, read and understand English, and own an Apple iPhone (5S and newer, running iOS 10.0 or higher) with an associated data plan or access to a home WiFi network. Potential participants unable to follow study procedures or with cognitive impairment that at the judgment of the investigators would preclude study participation were not eligible for enrollment.

### Procedures

PD participants had the FWC App installed on their own smartphone by study coordinators and were provided an Apple smartwatch. The devices were set up and synced in the clinic, and participants were provided contact information for technical support should they have encountered problems with the technology during the study period. Coordinators checked participant compliance weekly and reached out to participants who were not compliant with the study protocol with reminders or to provide troubleshooting assistance.

After a baseline assessment, in-clinic visits occurred at 2 weeks, 1, 3, and 6 months. During visits, participating clinicians first performed standard clinical assessments and then reviewed the study dashboard tool that dynamically displayed the data collected through the FWC App from each PD participant. Clinicians provided feedback on the data visualization and the utility of the dashboard during each in-clinic visit (Fig. 2 and Supplementary Table 1). Clinicians additionally participated in monthly focus groups with the CIS-PD study team and Intel Corporation to suggest iterative changes to the dashboard or highlight issues with the tool’s functionality. There were a total of 14 participating clinicians at the four sites. All clinicians were movement disorder neurologists. Prior to enrolling the first PD participant, clinicians participated in a study start up teleconference in which the process of how to use the clinician dashboard was explained.

### Study technology

As described above, the FWC App platform pairs a smartphone and smartwatch to collect both ePRO and sensor-derived data. The smartwatch used in this study was a commercially available Apple Watch Series 2 (with 38 mm display) with the following embedded sensors: tri-axial accelerometer, heart rate sensor, ambient light sensor, and gyroscope. For the purposes of this study, only accelerometer data were collected and stored in the study database. Participants were asked to wear the smartwatch for 12 h per day and at least 25 days per month.

In the FWC App, there was an interface for PD participants to view their activity level during the day and at night. The calculations used to derive these measures used a low-pass filter and summation of absolute acceleration in 5 and 30 s window intervals.^1^

The app also collected ePRO data, allowing PD participants to report and rate the symptoms they were currently experiencing (Supplementary Fig. 1). Each participant was asked to report their symptoms at least three times per day in the app. This included rating Constipation, Balance, Rigidity, Bradykinesia, Voice, Dyskinesia, Tremor, and Gait with response options of “none”, “slight”, “mild”, “moderate”, “severe”. These response choices were used so responses would correspond to the rating choices within the Movement Disorders Society - Unified Parkinson’s Disease Rating Scale (MDS-UPDRS). Each participant’s medication schedule was also added to the app at the beginning of the study and updated if any changes to their medication regimen were made. This schedule was used to trigger medication reminder alerts to PD participants and prompted them to respond with “taken” or “skipped” for each reminder.

### Statistical methods

Feasibility of remote capture wearable data was assessed by the retention of PD participants, the number of hours of data streamed, the number of PRO reporting instances (which could include one to eight different symptoms as described above) and the number of medication reports in the app relative to the expectation as per protocol. Average compliance was defined as an individual’s average of the following: (1) percent of expected hours streamed (12 h of streaming or 180,000 records per hour × 12 h = 2,160,000 records per day), (2) percent expected PRO reporting instances (three reporting instances per day), and (3) percent expected medication reports (based on an individual’s medication schedule). The tertiles of average compliance were used to defined “Low”, “Medium”, and “High” compliance. Compliance level was compared by demographic and baseline PD characteristics using either Kruskal–Wallis test or Fisher’s Exact test with two-sided *p*-values.

Clinical utility was assessed by asking whether clinicians were able to discern the data displayed in the study dashboard tool (“Yes” or “No”), and how these data assisted the clinician in their assessment (“Completely contradicted”/“Contradicted partially”, “Neither here nor there”, “Supported partially”/“Completely supported”). Reporting from the first version of the dashboard tool was compared with the final version of the dashboard tool, as it was iteratively developed over the course of the study.

This was an observational, exploratory study reporting descriptive statistics, thus no formal sample size calculations were done. An exploratory objective of the study was to provide initial validation of the use of mobile technology to capture ePROs versus in-office assessment via the MDS-UPDRS. The following ePROs reported in the app were compared with the corresponding MDS-UPDRS part 2 items: Balance Problems (MDS-UPDRS 2.12 Balance and Walking), Voice Problem (MDS-UPDRS 2.1 Speech), Tremor (MDS-UPDRS 2.10 Tremor). ePROs were averaged over the last week prior to the in-office visit and Spearman’s correlation coefficient and weighted Kappa coefficient were estimated for each visit.

### Reporting summary

Further information on research design is available in the Nature Research Reporting Summary linked to this article.

## Supplementary information

Supplementary Figure 1 and Table 1.

Reporting Summary

## Data Availability

Data is subject to third party (MJFF) restrictions and can be made available upon reasonable request to the corresponding author with permission of MJFF.

## Supplementary information

**Supplementary information** accompanies the paper on the *npj Digital Medicine* website (10.1038/s41746-019-0169-y).
