# Supplementary material for: Feasibility and utility of a clinician dashboard from wearable and mobile application Parkinson’s disease data
Source: NPJ Digit Med. 2019 Sep 25;2:95. doi: 10.1038/s41746-019-0169-y (PMC6761168; doi:10.1038/s41746-019-0169-y)
Supplement: Supplementary file 1 — Supplementary Figure 1 and Table 1. [file 41746_2019_169_MOESM1_ESM.pdf]

SUPPLEMENTARY TABLES AND FIGURES

- Supplementary Figure 1. Screen Shots of the *Fox Wearable Companion* mobile phone app as seen by PD patients
- Supplementary Table 1. Summary of Clinician Feedback for Dashboard Improvements obtained from Focus Group Sessions

Supplementary Figure 1. Screen Shots of the *Fox Wearable Companion* mobile phone app as seen by PD patients

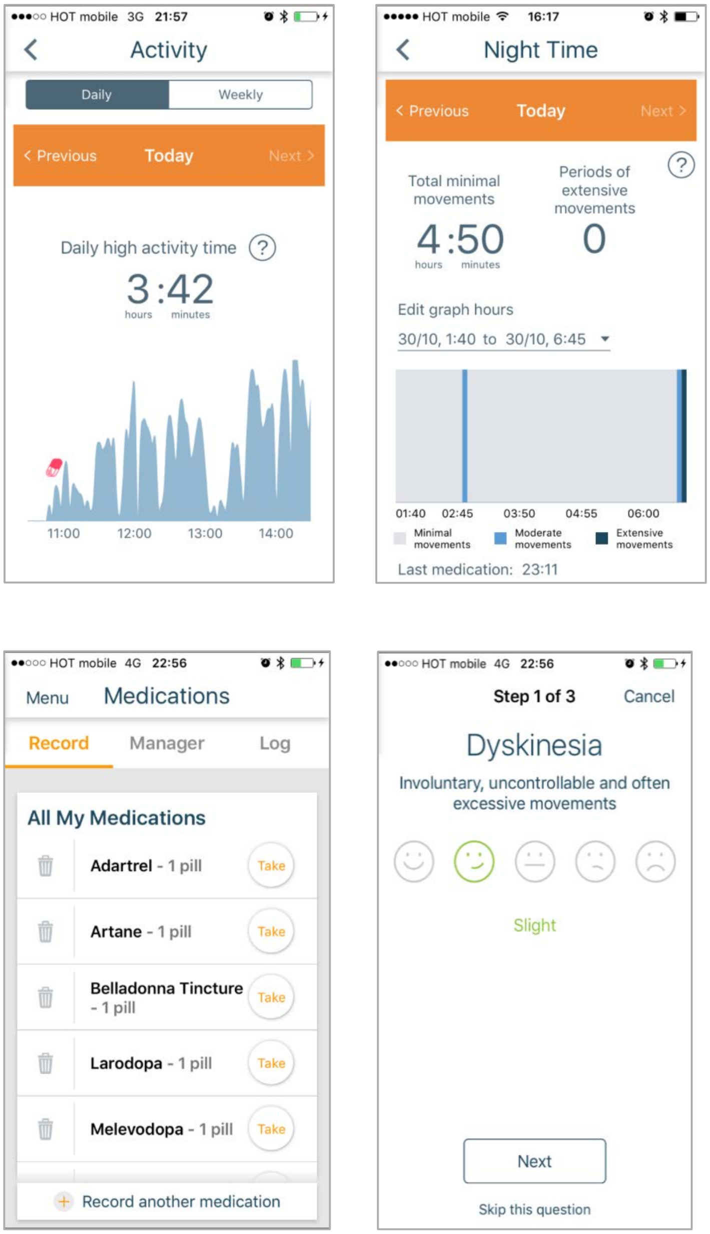

Supplementary Figure 1 Figure Legend: Screen shots of the App as seen by PD patients on their smartphone: Daily activity time, Periods of extensive, night time movements, Medication intake, and Dyskinesia severity.

Supplementary Table 1. Summary of Clinician Feedback for Dashboard Improvements obtained from Focus Group Sessions

**Activity Level Data Display (Sensor-Derived)**
- Utility in overlay of PRO reports (symptom reporting and medication intake) on intra-day activity level<br>- Utility in both high-level metrics on minutes of different activity levels in a day as well as a more granular minute-by-minute visual of activity level throughout a day.
**Sleep Activity Data Display (Sensor-Derived)**
- Need for patient reporting of sleep hours or use of algorithm to identify waking and sleeping hours.
**Symptom Reporting Data Display (PROs)**
- Utility in inter- and intra-day visuals of symptom reporting.<br>- Need for distinction between symptom report of 0 (zero, no symptom occurring) and missing report from patient.<br>- Utility in overlay of medication intake for intra-day symptoms reporting.<br>- Need for pop-up reminders for symptoms reporting.
**Medication Intake Data Display (PROs)**
- Need for patients to be able to edit medication intake in-app to ensure accuracy.<br>- Utility of clear Y-axis (axis showing hours of day) to ensure visual on hours of medication intake is clear for determining medication compliance.
**ON/OFF Diary Data Display (PROs)**
- Utility of clear Y-axis (axis showing hours of day) to ensure visual on hours of diary reporting is clear for determining ON/OFF time and symptoms.<br>- Utility in overlay of medication intake for contextualizing ON/OFF time and symptoms.
**General (Across All Data Displays)**
- Need for automatically adjusted time zone for location of participants and sites.<br>- Need for graph titles and descriptions to include clear information on HOW data is determined (PRO or sensor-derived, if sensor-derived, what metrics go into calculating) to ensure clinician decision making is informed.<br>- Utility in including non-motor symptoms in symptoms reporting.<br>- Utility in color-coding of medication intake markings for different classes of medications (e.g. dopamine agonists, etc).<br>- Utility in single visual with all data overlaid to provide “full” picture.
